# When too much isn’t enough: Does current food production meet global nutritional needs?

**DOI:** 10.1371/journal.pone.0205683

**Published:** 2018-10-23

**Authors:** Krishna Bahadur KC, Goretty M. Dias, Anastasia Veeramani, Clarence J. Swanton, David Fraser, Dirk Steinke, Elizabeth Lee, Hannah Wittman, Jeffrey M. Farber, Kari Dunfield, Kevin McCann, Madhur Anand, Malcolm Campbell, Neil Rooney, Nigel E. Raine, Rene Van Acker, Robert Hanner, Samantha Pascoal, Shayan Sharif, Tim G. Benton, Evan D. G. Fraser

**Affiliations:** 1 Department of Geography, Environment and Geomatics, University of Guelph, Guelph, Canada; 2 School of Environment, Enterprise and Development, University of Waterloo, Waterloo, Canada; 3 Department of Plant Agriculture, University of Guelph, Guelph, Canada; 4 Faculty of Land and Food Systems, The University of British Columbia, Vancouver, Canada; 5 Department of Integrative Biology, University of Guelph, Guelph, Canada; 6 Centre for Sustainable Food System, The University of British Columbia, Vancouver, Canada; 7 Canadian Research Institute for Food Safety, University of Guelph, Guelph, Canada; 8 School of Environmental Sciences, University of Guelph, Guelph, Canada; 9 Office of Research, University of Guelph, Guelph, Canada; 10 Department of Molecular and Cellular Biology, University of Guelph, Guelph, Canada; 11 Department of Pathobiology, University of Guelph, Guelph, Canada; 12 School of Biology, University of Leeds, Leeds, United Kingdom; 13 Arrell Food Institute, University of Guelph, Guelph, Canada; Wageningen University, NETHERLANDS

## Abstract

Sustainably feeding the next generation is often described as one of the most pressing “grand challenges” facing the 21^st^ century. Generally, scholars propose addressing this problem by increasing agricultural production, investing in technology to boost yields, changing diets, or reducing food waste. In this paper, we explore whether global food production is nutritionally balanced by comparing the diet that nutritionists recommend versus global agricultural production statistics. Results show that the global agricultural system currently overproduces grains, fats, and sugars while production of fruits and vegetables and protein is not sufficient to meet the nutritional needs of the current population. Correcting this imbalance could reduce the amount of arable land used by agriculture by 51 million ha globally but would increase total land used for agriculture by 407 million ha and increase greenhouse gas emissions. For a growing population, our calculations suggest that the only way to eat a nutritionally balanced diet, save land and reduce greenhouse gas emissions is to consume and produce more fruits and vegetables as well as transition to diets higher in plant-based protein. Such a move will help protect habitats and help meet the Sustainable Development Goals.

## Introduction

Producing enough food for the growing human population while reducing greenhouse gas (GHG) emissions and other environmental impacts from farming is a major global challenge [1–2]. Proposed solutions commonly focus on boosting production by approximately 70%, increasing yields in unproductive regions, eliminating waste, and reducing meat consumption [3–5]. Such solutions may also help reach some of the environmental targets set by international agreements such as the Paris Climate Agreement [6–7] and the Sustainable Development Goals (SDGs) [8–10]. To date, however, there has been no serious global evaluation of whether the production of different types of food (especially fruits and vegetables) is sufficient to provide a nutritionally balanced diet for the global population. Nor is it known whether a switch towards a nutritionally balanced diet might reduce the environmental impact of food production, thus helping meet SDGs and the Paris Agreement targets. A recent paper [11], however, found that a global shift towards current Western diets, an already observed trend in many parts of Asia, could lead to increased land use by 1 Giga hectare. This suggests that at least some commonly used nutritional guidelines need to be considered in terms of their impact on environmental sustainability [11]. Building on existing studies [12–14], this paper explores the extent to which global food production was nutritionally sufficient for 2011 (our baseline year when the world’s population was approximately 7 billion) and will be sufficient for a population of 9.8 billion, which is expected in 2050. We do this by comparing the types of diets nutritionists recommend versus global agricultural production statistics, and then explore options for producing a nutritionally balanced global diet.

## Data and methods

We begin by comparing the amount of food that is produced globally with what nutritional experts consider to be a healthy diet, and then estimate both the land use and greenhouse gas implications of switching to nutritionally recommended diets. To do this, we use a range of food and crop databases [15] along with different nutritional guidelines and recommendations [16–23] using the following assumptions.

### Choice of nutritional guidelines

While all nutritional guidelines are similar in that they recommend diets rich in fresh fruits and vegetables and low in sugars, different guidelines offer somewhat different advice regarding protein, dairy, starches, and grains. For instance, compared to the Harvard Healthy Eating Plate (HHEP) [18], the Canadian Food Guide (CFG) [17] suggests 27% fewer servings of fruits and vegetables, 34% fewer servings of meat/protein, but 60% more servings of dairy products and 25% more grains. Although some studies [24–28] show that the association between total fat/saturated fat and non-communicable diseases is mixed, there is a clear consensus across dietary guidelines that we should limit sugars, saturated and trans fats, oils and simple carbohydrates, and eat an abundance of fruits and vegetables. In addition, there is some speculation that nutritional food guidelines may be vulnerable to political and industry interference [29–30]. Given the controversies and discrepancies, in this study we opted to use the HHEP as it is a well-regarded nutritional guide that provides broadly consistent nutritional advice but is not linked with any particular national government or industry.

### Calculating actual and recommended servings

Diets are often described in terms of “servings” of different foods [17], but what constitutes a serving varies depending on the type of food. For instance, 125ml fresh or frozen vegetable is considered 1 dietary serving of vegetable, 1 slice of bread is considered 1 serving of grains and 75 g of cooked meat is considered 1 serving of protein. To calculate the actual number of dietary servings available worldwide, we used 2011 data from the United Nations’ FAO Food Balance Sheet [15] (S1 Table) and categorized the individual foods into the five broad food categories of the HHEP: whole grains, fruits and vegetables, protein, milk and oils. Given discrepancies in terms of what constitutes a fruit versus a vegetable we opted to combine fruits and vegetables into one single category. Finally, as sugar was not part of the HHEP diet, we considered it as a separate category.

Next, we determined an average number of calories per dietary serving for each type of food using guidelines from both the Canadian Food Guide [17] and the US Department of Agriculture [23]. Finally, we divided the available daily per capita calories for each food type by the number of calories per serving. This allowed us to calculate the number of available servings per person per day for each food type.

To calculate the number of servings needed to meet the HHEP requirements, we followed the following steps and assumptions. First, we interpreted the HHEP model as translating into the following recommendations: (1) 50% of our diet should be fruits and vegetables; (2) 25% should be whole grains; (3) the remaining 25% should be made up of protein, fat, and milk. Since there is considerable debate among nutritionists about specific levels of protein, fat and dairy, we assumed people following HHEP would consume: 1 serving of fat/oil, 1 serving of milk/dairy, and 5 servings of protein to make up this 25% of the diet. Given that assumptions had to be made, the calculations presented here represent only an approximation of the HHEP diet.

### Calculating the amount of land needed for existing vs. HHEP diets

FAO statistics provide a breakdown of the amount of food in each food category that is used for human consumption versus livestock feed. The statistics also provide a breakdown of the amount of protein produced by the dairy sector, by livestock in the form of meat, and by plants (see details in S2 Table). These statistics were used to calculate the amount of land used for each type of food, the amount of land devoted to livestock feed versus food for direct human consumption, and for meat versus dairy (Table 1). These calculations provided a baseline assessment of the amount of land used by these various types of agriculture for the year 2011, when the world’s population was approximately 7 billion people.

**Table 1:** Estimation of arable land area for milk and meat production*.

	Land area used to produce feed for livestock (million ha)	Land area used to produce feed for dairy (million ha) *	Land area used to produce feed for meat (million ha) *
**Whole Grains**	286.0	195.0	91.0
**Vegetables & Fruits**	19.4	13.2	6.2
**Oil Crops**	7.6	5.2	2.4
**Pulses**	9.9	6.7	3.2
**Total arable area for Livestock**	**322.9**	**220.1**	**102.8**

* Total milk production is 621.33 million litres and total meat production is 290.08 million kg according to FAO 2011 statistics

Next, we compared the amount of land currently devoted to these different food groups and the amount of land that would be required under the HHEP model using 2011 statistics. The surplus (or deficit) of land for each individual food group, as well as the total amount of the arable land needed, was then calculated to show how our demands for arable land would shift under the HHEP model. To account for the growing human population, we extrapolated food production and land-use requirements using the United Nations’ mid population projection of 9.8 billion by 2050. To account for rising technological sophistication, we assumed a 1% annual increase in yield that corresponds to historic patterns in yields in FAO statistics. Finally, we estimated the impacts of adopting a HHEP diet on the total amount of arable land and the total amount of pasture land today and in the future, following the FAO definition of pasture land as “…land used permanently (five years or more) for herbaceous forage crops, either cultivated or growing wild …” (FAO 2018, page 2)[31].

### Calculating greenhouse gas emissions

We used a life cycle approach to calculate GHG emissions for different types of food by multiplying a food’s GHG emission factor (in carbon dioxide equivalents per kg of food) times the mass of annual global production of that food type. GHG emission factors were obtained from a database developed by Veeramani et al. [32]. Calculations were performed using SimaPro Lifecycle Assessment (LCA) software [33]. GHGs are calculated to the farm gate and include raw material extraction for agricultural inputs such as fertilizer and fossil fuels, but they do not include GHG emissions from land use change or soil carbon sequestration. The GHG estimates are meant only to provide trends related to changes in diets. Available life cycle studies and databases for foods produced under the range of conditions that occur globally [34] are limited, therefore, some emission factors are based on global data, while others come from European sources. Furthermore, GHG emissions from land use change and management, and resulting changes in soil carbon or biomass cover, are not generally included in these databases. As a result, these estimates are mostly useful for looking at changes in the direction of emissions rather than providing an accurate assessment of the absolute amounts of GHGs emitted for different food products.

A global emission factor for fish was estimated based on global fishing fleet fuel consumption [35], since this is the main source of GHG emissions in the wild-caught fish supply chain. Fish from aquaculture operations was not considered due to lack of globally representative data on this system. GHG emissions associated with cattle production depend on how the cattle are raised, so an average emission factor was developed to approximate both “best” and less efficient management practices in the major cattle-producing countries [36–37]. It was assumed that 50% of cattle were under best management and 50% under conventional and less efficient practices. This is likely to underestimate emissions as more than 50% of the world’s cattle are located in Brazil or India [38], where practices are still relatively inefficient.

Finally, as emission factors in the LCA databases are based on live weight, the following conversions were used to relate carcass weight to live weight: 52% for bovine meat, 56% for sheep and goat, 72% for poultry [39], and 50% for fish [40]. The LCA databases used to obtain GHG emissions do not include all the food types reported in the FAO food balance sheets. Therefore, whenever a food type was missing, the mass of that food type was redistributed amongst the available food items. For example, edible offal and mutton/goat are not listed in the LCA database, so the amount of these foods was redistributed amongst the available animal products.

The annual consumption, on a mass basis, of each food for two population sizes, and HHEP versus current diet, was determined based on an existing methodology [16] modified to consider the number of servings required to meet the HHEP diet. Furthermore, we set the proportions of the different food items in the HHEP diet to match the proportions in the FAO production data; for example, if beef was 50% of total animal protein in the FAO 2011 production statistics, we maintained the same ratio in the HHEP diet even though HHEP recommends red meat only 2 times per week. Hence (as discussed below) the analysis overestimates the impact of meat because it retains current proportions of red meat. While the inclusion of fish in the diet does not affect land use patterns, it does have significant implications for GHGs. However, nutritional recommendations for the ideal amount of fish differ, with the HHEP recommending 1–2 servings of high-omega content fish per week [41] and the University of Michigan’s dietary recommendations suggesting 2–4 servings [42]. Here, we used 2 servings per week, which translated to 9% of the required protein servings.

Overall, these assumptions introduce some uncertainty in the absolute value of the GHG emission calculations. Nevertheless, this approach provides useful relative values for the purpose of comparisons.

### Scenario analysis

Finally, to provide a rough estimate of the implications of different possible strategies, we estimate the impact of four possible future scenarios:

- A scenario where all livestock consumption is replaced by plant-based proteins;
- A scenario where consumers reduce livestock consumption to 20% of their protein (consistent with the current ratio of meat: plant-based protein in India);
- A science and technology scenario where new technologies increase crop yields;
- A household food waste reduction scenario.

### Assumptions and Limitations

To assess some of the implications of moving towards a nutritionally balanced global diet, we made several assumptions which need to be considered in the interpretation of the results:

- HHEP provides general guidance rather than specific recommendations. Therefore, the results presented here are based on our interpretation of the HHEP model.
- To estimate the amount of land required for each food type, we assumed a constant ratio of food and feed. In reality, however, the amount of land used to produce food and feed, including that used to produce different types of livestock, varies depending on geography, production systems, etc. [43].
- We had to assume that land currently used for cereal, sugar and oil production can be switched to fruit, vegetable and protein crop production. Although land used for cereals and oils can likely be used for plant protein crops (e.g., producing soy on land currently used for maize), some vegetables and fruits are likely to require different agro-climatic conditions.
- Based on FAO’s historic data showing that crop yields have increased by approximately 1% / year on average, we assumed that this trend will continue due to technology adoption for each crop in every part of the world. In reality, yield increases are likely to be more variable due to factors such as climate change and other unforeseen changes to the agricultural system.
- Finally, since the FAO data include farm to retail waste but do not include household waste, we used a global average of 20% household-level food waste based on averaging estimates from Gustavsson *et al*. [44]. However, this represents both avoidable and unavoidable waste, and there are no data to determine how much of this is avoidable.

## Results

### Comparison of available vs. healthy food scenarios

Currently, worldwide food production exceeds 2,750 kilocalories per person per day [15], which exceeds the amount required to feed the global population. Although these data account for farm-level waste, they do not include the estimated 20% household food waste [44]. Hence, currently available calories are likely to be about 2,200 kilocalories per person per day, which is sufficient for the world’s current population [23].

**Fig 1:**
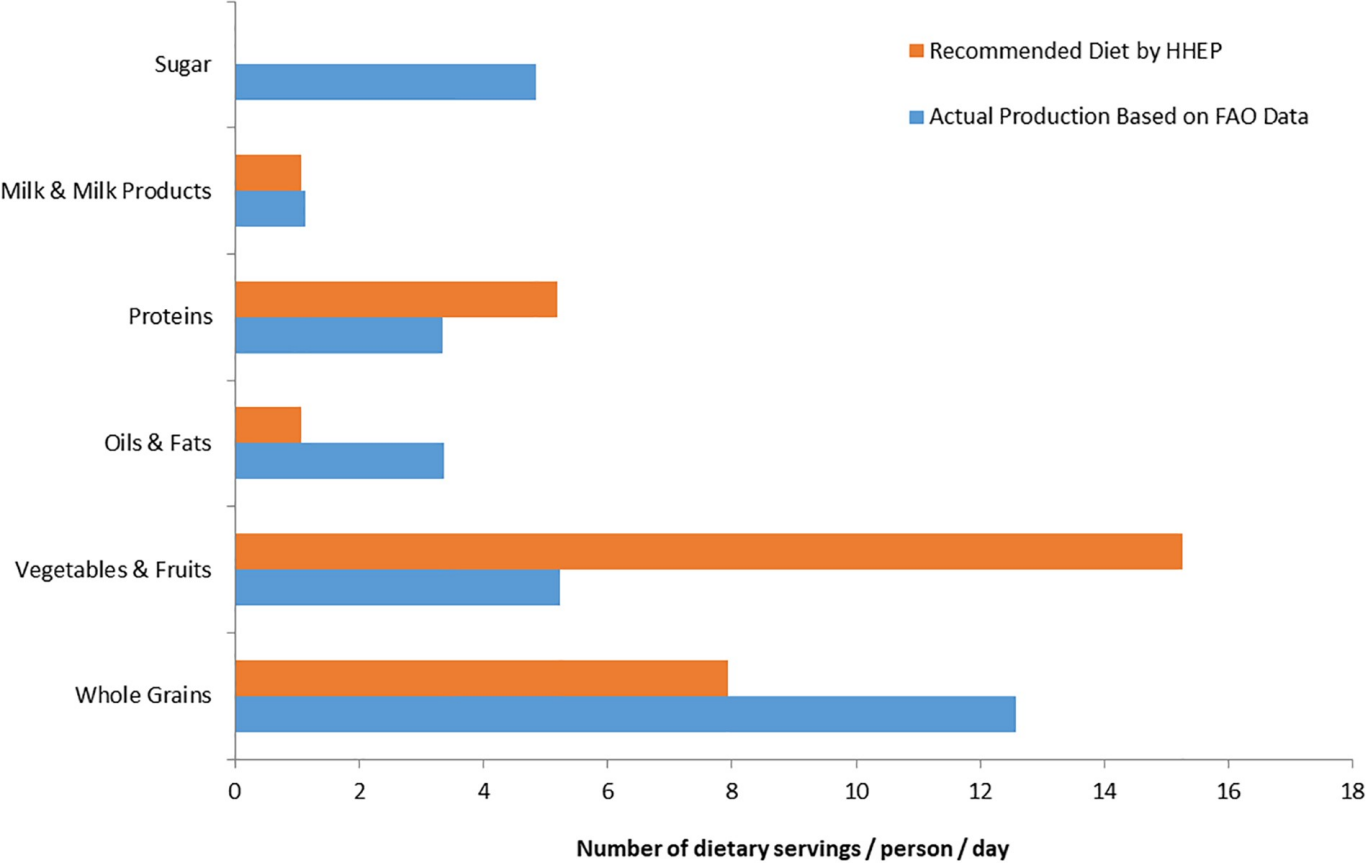
Global production versus recommended consumption. Global food production (blue bars) are from FAO (2011) data and nutritional recommendations (orange bars) are based on Harvard University Healthy Eating Plate model. All data are displayed in dietary servings following the CFG [17] and USDA guidelines [23].

However, when global production is divided into different food groups, a radically different picture emerges. Specifically, global agriculture currently produces 12 servings of grains, 5 of fruits and vegetables, 3 of oil and fat, 3 of protein, 1 of milk and 4 servings of sugar per person per day (Fig 1). In contrast, using the HHEP, we estimate that global agriculture production should provide 8 servings of whole grains, 15 servings of fruits and vegetables, 1 serving of oil, 5 servings of protein, and 1 serving of milk per person per day to provide a nutritionally balanced diet (Fig 1). Thus, the world currently over-produces grains, fats, and sugars while greatly under-producing fruits and vegetables and, to a smaller extent, proteins (Fig 1).

Within the broad food categories, there are further mismatches between current production and recommended consumption. While the HHEP model suggests that total fruit and vegetable servings should comprise about 40% fruit and 60% vegetables, fruits currently make up only 28% of global fruit and vegetable production. Moreover, of global vegetable production, almost 40% consists of starchy vegetables such as cassava and potatoes, which, according to sources such as the HHEP and Diabetes Canada, should not count as vegetable servings because of their effect on blood sugar. In addition, almost two-thirds of animal protein consumed is red meat that the HHEP model regards as the least desirable protein for human health and generates more greenhouse gases than other proteins [45].

### Land use implications

Our analysis found that if global agriculture produced foods at the levels recommended by the HHEP then the amount of arable land devoted to:

- Grain production would be reduced by 150 million ha;
- Fat and oil production would drop by 105 million ha; and
- Sugar production would drop by up to 30 million ha.

At the same time, the arable land devoted to fruits and vegetables would need to increase by 171 million ha. To meet our needs for protein, the amount of arable land devoted to livestock feed would increase by 57 million ha while land used for plant protein would increase by 20 million ha. Overall, therefore, shifting production to match the diet recommended by the HHEP would reduce the amount of arable land needed for agriculture by a total of 51 million ha (Table 2). This reduction in agricultural land use could help global efforts to conserve biodiversity and enhance ecosystem services, thus helping meet the Sustainable Development Goals.

**Table 2:** Land area (in million ha) using FAO data, assuming universal adoption of the Harvard Healthy Eating Plate (HHEP) nutritional guidelines for 7 billion people (today) and 9.8 billion people (projected for 2050) assuming that the yields of all crops continue to grow by 1%/year following historic trends. Percentage change from current values is given for alternative scenarios in parentheses.

Food groups	Without adoption of HHEP (existing diet)		With adoption of HHEP diet					
			Existing ratio of protein from livestock and plants		20% Protein from animal sources and 80% protein from plants sources		Protein from plants only	
	For 7 billion (FAO data today)	For 9.8 billion (2050)	For 7 billion (today)	For 9.8 billion (2050)	For 7 billion (today)	For 9.8 billion (2050)	For 7 billion (today)	For 9.8 billion (2050)
Whole grains	407	411	257 (-37%)	260 (-36%)	257 (-37%)	260 (-36%)	257 (-37%)	260 (-36%)
Fruits & Vegetables	89	90	260 (+192%)	263 (+196%)	260 (+192%)	263 (+196%)	260 (+192%)	263 (+196%)
Oils & Fat	153	155	48 (-69%)	49 (-68%)	48 (-69%)	49 (-68%)	48 (-69%)	49 (-68%)
Livestock Protein	103	104	160 (+55%)	162 (+57)	39 (-62%)	40 (-61%)	0	0
Plant Protein	36	37	56 (+56%)	57 (+58)	267 (+642%)	270 (+650%)	334 (+828%)	338 (+839%)
Milk/dairy	220	222	206 (-6%)	208 (-5%)	206 (-6%)	209 (-5%)	206 (-6%)	208 (-5%)
Sugar	30	31	0	0	0	0	0	0
**Arable land Total**	**1038**	**1050** **(+1%)**	**987** **(-5%)**	**999** **(-4%)**	**1077** **(+4%)**	**1091** **(+5%)**	**1104** **(+6%)**	**1118** **(+8%)**
Pastureland for Meat	1092	1529	1699 (+56%)	2377 (+118)	409 (-63%)	573 (-48%)	0	0
Pastureland for Milk/dairy	2341	3277	2192 (-6%)	3073 (+31%)	2192 (-6%)	3069 (+31%)	2192 (-6%)	3073 (+31%)
**Pastureland total**	**3433**	**4806** **(+40%)**	**3891** **(+13%)**	**5450** **(+59%)**	**2601** **(-24%)**	**3642** **(+6%)**	**2192** **(-36%)**	**3073** **(-10%)**
**Grand total**	**4471**	**5856** **(+31%)**	**4878** **(9%)**	**6449** **(+44%)**	**3678** **(-18%)**	**4733** **(+6%)**	**3296** **(-26%)**	**4191** **(-6%)**

The situation is different when we consider pastureland in addition to arable land. As noted in Table 2, 3,433 million ha of pastureland are currently used to raise livestock. Increasing protein production to levels consistent with the HHEP recommendations would require 3,891 million ha. This underlines the need for greater reliance on other protein sources [1–3].

For an estimated 9.8 billion global population by mid-century, our analysis shows that, if diets remain static and farming continues to produce the same proportions of food as it does today, we will require 12 million ha more arable land and 1,373 million ha more pasture land. With universal adoption of the HHEP diet, global agriculture would need 39 million ha less arable land and 2,017 million ha more pasture land. If we combine universal adoption of the HHEP diet with a diet where only 20% of protein comes from livestock, then global agriculture would need 53 million ha more arable land and 209 million ha more pastureland. By contrast, with a complete shift to a vegetarian diet where protein comes from leguminous crops, global agriculture would need 80 million ha more arable land and 360 million ha less pasture land to feed the world’s 2050 population (Table 2). This scenario is unrealistic (as discussed below) but is presented for purpose of comparison.

### Greenhouse gas emission impacts

Using statistics from our baseline year of 2011, we estimate that adopting the HHEP diet will increase total cradle-to-farm-gate GHGs relative to the world’s existing diet by approximately 2.8 GT of CO_2_e/year or by 49%, exclusive of any additional GHGs that could occur due to land use change (Table 3). This is highly problematic since to meet the goal of keeping global mean annual temperature within 1.5 degrees Centigrade of pre-industrial levels, humanity can emit only ~200 GT more CO_2_ [46] and implementing the HHEP would use up this GHG allowance in 70 years. However, most of this rise in GHGs would be due to increasing the amount of animal-source protein, which contributes over 50% of the GHG emissions under the current scenario but increases to 70% in the HHEP scenario (S3 Table). By contrast, plant protein (from legumes, seeds and nuts) contributes only 3% of the total GHGs, and fish contributes only 4%.

**Table 3:** Greenhouse gas emissions (GT CO2e/yr) using FAO data, assuming universal adoption of the Harvard Healthy Eating Plate (HHEP) nutritional guidelines for 7 billion people (today) and 9.8 billion people (projected for 2050). Percentage changes from the current values are given for both alternative scenarios in parentheses.

Food groups	Without adoption of HHEP (Existing diet)		With adoption of HHEP diet			
			Existing ratio of protein from livestock and plants		Protein from plants only	
	For 7 billion (FAO data today)	For 9.8 billion (2050)	For 7 billion (today)	For 9.8 billion (2050)	For 7 billion (today)	For 9.8 billion (2050)
Whole grains	0.88	1.24	0.54 (-39%)	0.76 (-14%)	0.54 (-39%)	0.75 (-15%)
Fruits & Vegetables	0.32	0.44	0.58 (+81%)	0.82 (+156%)	0.58 (+81%)	0.81 (+153%)
Oils & Fat	0.07	0.10	0.03 (-57%)	0.04 (-43%)	0.03 (-57%)	0.04 (-43%)
Livestock Protein	2.90	4.06	5.85 (+102%)	8.19 (+182%)	0	0
Fish Protein	0.38	0.53	0.34 (-11%)	0.47 (+24%)	0	0
Plant Protein	0.12	0.17	0.22 (+83%)	0.30 (+150%)	1.58 (1217%)	2.21 (1742%)
Milk/dairy	0.64	0.89	0.59 (-8%)	0.83 (+30%)	0.59 (-8%)	0.84 (+31%)
Sugar	0.04	0.05	0	0	0	0
**GHGs Total**	5.64*	7.89** (+40%)	8.39*** (+49%)	11.74**** (+108%)	3.56*** (-37%)	4.99**** (-12%)

*Includes 0.29 GT of CO2e/y for transportation and upstream energy use

**Includes 0.41 GT of CO2e/y of for transportation and upstream energy use

*** Includes 0.24 GT of CO2e/y for transportation and upstream energy use

**** Includes 0.34 GT of CO2e/y for transportation and upstream energy use

We also considered what would happen if the HHEP diet were implemented with high animal protein (at current levels), but with only 2 servings of red meat per week. In this case, the total GHG emissions would increase by only 0.7 GT from current levels (S3 Table). These data suggest that pescetarian or vegetarian diets could result in decreasing GHG emissions (Table 3 and S3 Table). The implications of this will be discussed below.

## Discussion: Three pathways for future diets

Our primary finding is to illustrate the fundamental mismatch between what global agriculture produces and what the world’s population requires for a balanced diet as recommended by nutrition experts. Although global agriculture already produces enough calories for the world’s current population, there is insufficient production of fruits, vegetables and protein and major over-production of energy-dense foods, especially sugars, cereals and oils. Consequently, people must over-consume these products in order to meet their calorie requirements. The failure of global agriculture to provide a balanced diet presumably contributes to the current epidemic of obesity and diabetes [47].

The analysis also quantifies the land use and GHG effects if diets remain static and farming continues to produce the same proportions of food as it does today for the projected 2050 population of 9.8 million. Briefly, if nothing else changes, the data suggest we will require 12 million ha more arable land and 1,373 million ha more pastureland and produce 2.25 GT more GHGs annually. In contrast, with universal adoption of the HHEP diet, global agriculture would need 1,978 million ha total land and produce 6.15 GT more annual GHGs (Tables 2 and 3). As these estimates exceed the available land base and acceptable emissions, we consider three potential pathways for the future.

### Pathway 1: A shift to proteins that require less land and produce fewer GHGs

One way to reduce the GHGs associated with our diets would be to both improve the efficiency of livestock systems and reduce the proportion of protein we obtain from animal agriculture (see Table 3). With that said, this analysis should not be seen as a rationale for a purely vegetarian diet. Livestock plays an important role in many agro-ecosystems, 987 million people worldwide depend on raising animals as a key livelihood strategy [48–49], and much pasture land is ill suited to crop production. In parts of the world where malnutrition is still prevalent, increased consumption of livestock products can help improve the well-being of the rural poor [48–49]. In addition, animal agriculture and animal-based diets are culturally important for people around the world [50]. Hence, meat consumption will continue, but cannot persist at today’s levels without major consequences.

Overall, therefore, the data suggest that the environmental footprint of food and farming systems would drop with increased reliance on plant-based or alternative proteins such as fungus, algae or insects [51]. In addition, scientific work is currently helping improve the efficiency by which animals convert feed into useable meat [52] and to breed animals (especially cattle) that produce fewer GHGs [53]. It is also possible to reduce the environmental impact of livestock and increase soil carbon sequestration through approaches such as “high-density short rotation” grazing where relatively high numbers of cattle are placed on a small pasture for a short period [54].

**Fig 2:**
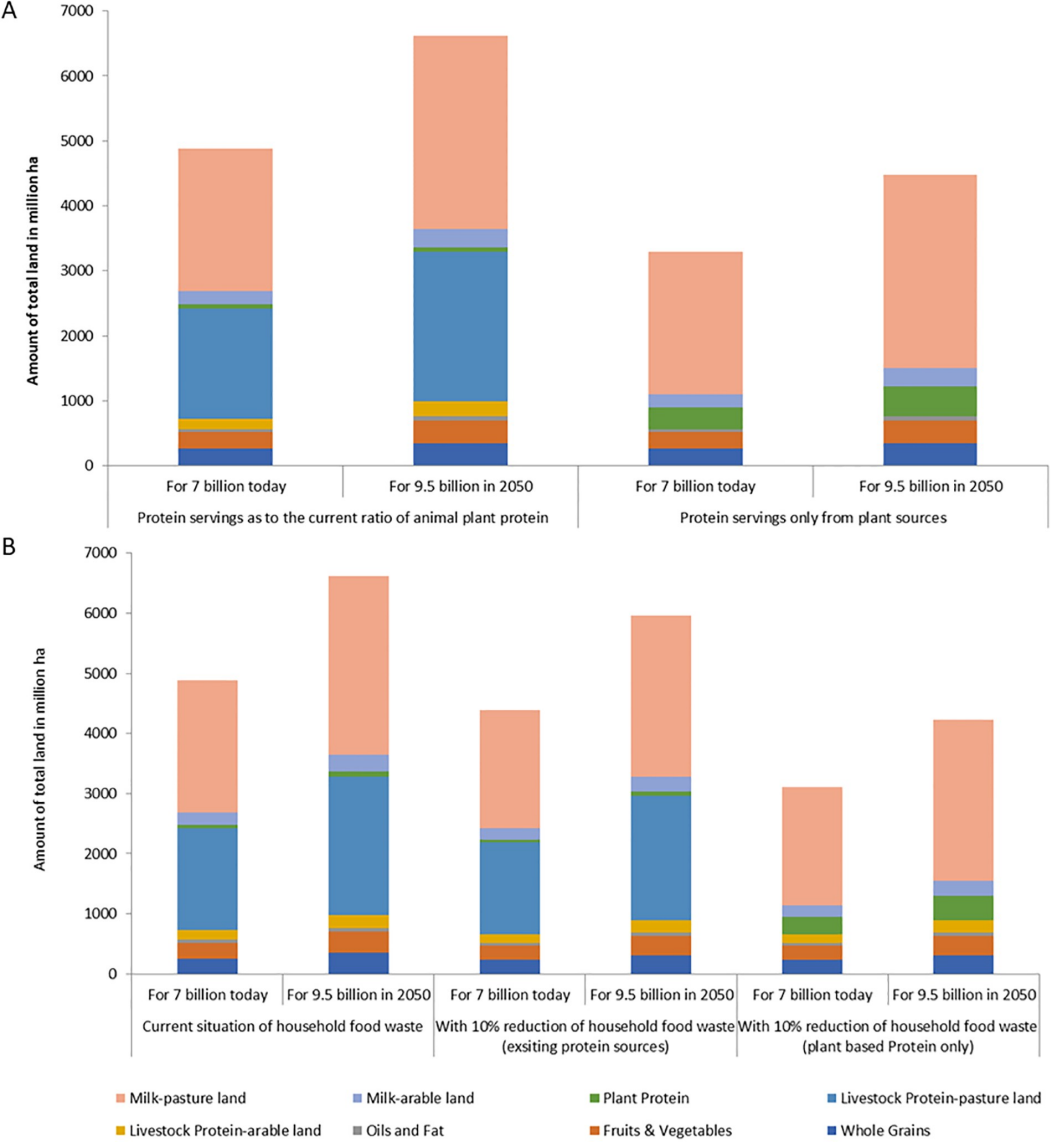
Comparison of the amount of land used in million ha, with the adoption of HHEP diet for 7 billion people (today) and 9.8 billion people (in 2050): A) For protein servings from animal and plant sources and plant sources only (Upper panel). B) With the current situation of household food waste and with 10% household food waste (Lower Panel).

The situation is more complex regarding land use. As noted above, even with universal adoption of the HHEP diet, producing enough protein for the global human population will increase the amount of arable land associated with farming due to the need to expand the production of high-protein leguminous crops (Table 2, Fig 2A). This could have serious environmental consequences relating to soil loss, the GHGs emitted from land use change, and the biodiversity loss associated with commodity farming [55]. However, reducing the amount of pastureland used to raise livestock may compensate for this increase in arable farming. Therefore, when both arable and pasture lands are considered (see Table 2), the data show that the total amount of land used by agriculture would not have to rise if there was a shift to both the HHEP and a much greater consumption of plant-based protein (Fig 2A). This finding is consistent with the conclusions of Rizvi et al. [11] who showed that current Western dietary guidelines, which rely on non-plant-based proteins, would not be sustainable if adopted globally.

Increasing fish consumption is another useful strategy as fish represent a relatively low GHG emitting supply of protein and have been found to improve heart and blood vessel health and reduce cardiovascular disease [41]. Nevertheless, there are other concerns including contamination of fish by toxins, the overfishing of oceans, and habitat loss [41,56,57]. Hence, more effort is needed to develop sustainable sources of fish (perhaps through improved and land-based aquaculture or aquaponics) and promote sustainable fishing practices [58–59].

Overall, the analysis presented here reinforces the growing number of studies that demonstrate the importance of developing new protein systems (based on plants, algae, fungi or insects) that require a smaller land base and fewer resources. Such systems, along with improvements in more conventional livestock systems, will be needed in the future to maintain adequate protein production without destroying our ability to meet either SDGs or the Paris Agreement targets.

### Pathway 2: Science and technology to increase yields

A second approach would be to use science and technology to increase yields, especially for fruits and vegetables, of which many are pollinator-dependent [60–61]. In the calculations above, we assumed that technological advancements will continue to increase yields by 1% per year which is consistent with how technology has increased yields over the past 50 years. However, a switch to HHEP, plus the rising demand due to population growth, would require a yield increase of 8% per year for fruits and vegetables. This would represent a huge scientific and technological challenge and would require a realignment of international agri-food research away from the current primary focus on cereals and starchy foods [62] towards research on fruits and vegetables. Similarly, to maintain HHEP-recommended levels of protein and dairy without increasing land requirements, yields would need to increase by 3% per year for meat-based protein and by 0.8% per year for dairy (Table 4).

**Table 4:** The annual percentage yield increases needed to produce the HHEP diet for the world population of 2050 without an increase in the amount of arable land.

Food Groups	9.8 Billion population by 2050
Whole Grains	No increases needed*
Fruits & Vegetables	8.72%/yr
Oils	No increases needed*
Protein	3.27%/yr
Milk/dairy	0.79%/yr
Sugar	No increases needed*

* Since we are currently producing grains, oils & fats and sugars in excess of projected need, these categories can either be less intensively farmed or land could be taken out of production for these crops, thus reducing agriculture’s impact on ecosystem services.

Increasing production of fruits and vegetables, without increasing the amount of arable land used by agriculture, might also be achieved in part through urban agriculture, innovations in vertical farming, indoor production facilities driven by LED lighting and hydroponics, and other advanced horticultural production technologies [63,64]. Such innovative production systems must be developed in tandem with farmer-directed, participatory plant breeding and genetics programs, and support for pollination services, in order to boost yields.

### Pathway 3: Reducing waste

Another possible solution could be a reduction of household food waste. Given the FAO data include farm-to-retail waste, but do not include household waste that is estimated to be as high as 20% [44], we calculated how halving this amount of household waste would affect both arable and pasture land requirements for both current diets and for a diet using only plant-based protein. If global household food waste were reduced by half, then instead of requiring 987 million ha of arable land for 7 billion people and 999 million ha for 9.8 billion people, requirements would be only 888 and 899 million ha respectively. Similarly, when we consider a future *both* that increases the consumption of plant-based protein *and* reduced waste, the requirement for pasture land should be only 1,973 million ha for 7 billion people and 2,763 million ha for 9.8 billion compared to the current 3,433 million ha of pasture land (Fig 2B). In terms of GHG emissions, reducing waste from 20% to 10% would reduce resource use for food production, thus reducing emissions by 10% (in addition to reducing methane emissions from landfilling).

## Conclusions

In summary, (1) current agricultural production fails to provide the mixture of foods needed for the world’s population to have the type of balanced diet recommended by nutritionists; (2) rectifying this imbalance would save arable land, but (3) also saving pasture land and reducing GHG emissions would require more reliance on plant-based sources of protein. Furthermore, if the world’s population grows as anticipated, food production must change to fit within available land and acceptable levels of land use and GHG emissions [11, 65–66]. The data suggest that adopting nutritionally balanced diets that involve a greater consumption of fruits and vegetables, plus lower consumption of grains, fats and sugars, along with developing proteins that require less land to produce should help to ensure sustainable and balanced diets through the coming decades [e.g. see: 67]. Such a transition would reduce global GHG emissions, better support ecosystem services and biodiversity, and have significant benefits for human health.

## Supporting information

S1 Table Available Kilocalories and their equivalent servings from the FAO’s Food balance sheets for agricultural year 2011. Serving calculations were based on Canada’s Food Guide serving sizes and USDA guidelines.
(PDF)

S2 Table The amount of food produced, their uses for human food and livestock feed and arable land area under each food group based on FAO 2011 data_._ (PDF)

S3 Table Breakdown of greenhouse gas emissions by food category. (PDF)
