# Supplementary material for: When too much isn’t enough: Does current food production meet global nutritional needs?
Source: PLoS One. 2018 Oct 23;13(10):e0205683. doi: 10.1371/journal.pone.0205683 (PMC6198966; doi:10.1371/journal.pone.0205683)
Supplement: S1 Table — Serving calculations were based on Canada’s Food Guide serving sizes and USDA guidelines. (PDF) [file pone.0205683.s001.pdf]

Table S1. Available Kilo-calories and their equivalent servings from the FAO's Food Balance Sheets for agricultural year 2011. Serving calculations were based on CFG and USDA guidelines.

**Whole Grains**	**Kcl/ Week**	**Kcl/ Serving**	**# of servings**
Wheat & products	3668	110	33.34
Rice & products	3815	108	35.30
Barley & Products	49	97	0.50
Maize & Products	1015	72	14.00
Rye & Products	42	89	0.50
Oats	21	120	0.17
Millet & Products	189	104	1.80
Sorghum & Prod	217	109	2.00
Cereals, Other	49	120	0.40
Total/week	9065		88.01
**Total/day**	**1295**		**12.57**

**Sugar & Sweeteners**			
Sugar, Non-Centrifugal	56	42	1.33
Sugar, Raw	1326	45	28.70
Honey	14	64	0.23
Sweeteners, Other	161	54	3.00
Sugar Cane	28	45	0.66
Total/week	1585		33.92
**Total/day**	**226**		**4.85**

**Vegetables and Fruits**	**Kcl/ Week**	**Kcl/ Serving**	**# of servings**
**Vegetables**			
Cassava & Products	266	160	1.60
Potatoes & Products	448	68	6.60
Sweet Potatoes	147	90	1.60
Yams	84	79	1.00
Roots, Other	42	40	1.00
Onions	84	32	2.60
Tomatoes & Prod	70	16	4.40
Vegetables, Other	497	75	6.80
**Fruits**			
Oranges	70	35	2.00
Lemons & Prod	7	60	0.10
Grapefruit	7	40	0.20
Citrus	7	70	0.10
Bananas	140	110	1.00
Plantains	56	100	1.00
Apples	77	105	0.75
Pineapple	21	41	0.50
Dates	28	75	0.38
Grapes	42	41	1.00
Fruits, Other	203	50	4.00
Total/week	2296		36.63
**Total/day**	**328**		**5.23**

**Fat and oil**	**Kcl/ Week**	**Kcl/ Serving**	**# of servings**
**Oil crops**			
Soybeans	98	100	1.00
Coconuts	77	71	1.00
Sunflower Seeds	7	204	0.06
Sesame Seeds	21	206	0.20
Olives	7	150	0.07
**Vegetable Oils**			
Soybeans	595	120	5.00
Sunflower	231	124	1.86
Rape & Mustard	252	124	2.00
Cottonseed	98	120	0.82
Palm kernel	42	117	0.36
Palm	357	120	3.00
Coconut	49	117	0.42
Sesame seed	21	120	0.17
Olive	77	119	0.65
Rice bran	21	120	0.17
Maize Germ	56	120	2.20
Other	42	120	0.35
**Animal Fats**			
Butter, Ghee	203	100	2.00
Cream	14	60	0.25
Raw	210	100	2.00
Total/week	2478		23.57
**Total/day**	**354**		**3.37**

**Meat and alternatives**	**Kcl/ Week**	**Kcl/ Serving**	**# of servings**
**Meat**			
Bovine Meat	280	140	2.00
Mutton & Goat	77	150	0.50
Pork	840	120	7.00
Poultry	399	160	2.50
Edible Offal	49	120	2.40
**Fish/Seafood**			
Freshwater	91	100	1.00
Demersal	42	85	0.50
Pelagic	56	140	0.40
Crustaceans	14	84	0.17
Cephalopods	7	123	0.17
Molluscs, Other	7	105	0.20
Aquatic Plants	14	23	0.60
Eggs	245	130	2.00
Beans	161	161	1.00
Peas	56	173	0.33
Pulses, Other	238	202	1.00
Nuts & Products	266	166	1.60
Total/week	2842		23.37
**Total/day**	**406**		**3.34**

**Milk & Products**	973	120	8.00
Total/week	973		8.00
**Total/day**	**139.0**		**1.14**
