# Supplementary material for: When too much isn’t enough: Does current food production meet global nutritional needs?
Source: PLoS One. 2018 Oct 23;13(10):e0205683. doi: 10.1371/journal.pone.0205683 (PMC6198966; doi:10.1371/journal.pone.0205683)
Supplement: S2 Table — (PDF) [file pone.0205683.s002.pdf]

Table S2. The amount of food produced, their uses for human food and livestock feed and arable land area under each food group based on FAO 2011 data.

Whole Grains	Area harvest (M ha)	For Food (M ton)	For Feed (M ton)	Area for food (M ha)	Area for feed (M ha)
Wheat	220.3	446.9	141.0	167.5	52.8
Rice	162.5	370.3	33.0	149.2	13.3
Barley	48.5	6.5	88.2	3.3	45.2
Maize	171.7	120.5	485.1	34.2	137.5
Rye	0.5	5.7	4.8	0.3	0.2
Oats	9.6	4.0	15.5	2.0	7.6
Millet	33.8	22.9	3.9	28.8	4.9
Sorghum	42.1	25.2	26.4	20.6	21.5
Cereals other	3.7	5.4	18.2	0.9	2.8
**Total**				406.7	286.0
**Fat and Oil**					
Soybeans	103.8	234.2	13.3	98.2	5.6
Coconuts	12.0	2.0	0.0	12.0	0.0
Sun flower seeds	25.7	32.4	2.7	23.7	1.9
Sesame seed	8.6	4.0	0.0	8.6	0.0
Rape & mustard	10.1	3.2	0.0	10.1	0.0
Olives	0.7	58.2		0.7	0.1
**Total**				153.3	7.6
**Sugar**					
Sugar cane	25.6	34.0		25.6	
Sugar beets	5.1	16.9		5.1	
**Total**				30.6	

Vegetables and Fruits	Area harvest (M ha)	For Food (M ton)	For Feed (M ton)	Area for food (M ha)	Area for feed (M ha)
Cassava	20.6	100.7	77.6	11.6	9.0
Potato	19.3	237.2	49.7	15.9	3.3
Sweet potato	8.2	54.6	39.9	4.8	3.5
Yams	6.5	29.1	8.2	5.1	1.4
Roots other	1.3	13.2	0.7	1.2	0.1
Onions	4.5	76.8	0.1	4.5	0.0
Tomatoes & Prod	4.7	138.5	1.8	4.7	0.1
Vegetables, Other	18.5	714.7	50.8	17.3	1.2
Oranges	4.0	91.3	0.0	4.0	0.0
Bananas	5.1	82.5	1.5	5.0	0.1
Grapefruit	0.3	7.4	0.0	0.3	0.0
Citrus	1.3	10.0	0.0	1.3	0.0
Lemons & Prod	1.0	13.0	0.0	1.0	0.0
Plantains	5.4	24.1	2.9	4.8	0.6
Apples	5.0	64.4	0.6	4.9	0.0
Pineapple	1.0	19.6	0.0	1.0	0.0
Dates	1.1	6.2	0.3	1.0	0.0
**Total**				88.4	19.4
**Pulses**	46.0	47.2	12.9	36.1	9.9
**Meat**		290.1			
**Milk**		621.3			
