# Supplementary material for: When too much isn’t enough: Does current food production meet global nutritional needs?
Source: PLoS One. 2018 Oct 23;13(10):e0205683. doi: 10.1371/journal.pone.0205683 (PMC6198966; doi:10.1371/journal.pone.0205683)
Supplement: S3 Table — (PDF) [file pone.0205683.s003.pdf]

Table 3. Breakdown of Greenhouse Gas Emissions by food category

Category	FAO 2011	FAO 2050	HEP-High# Animal Protein*, high red meat 2011	HEP-High Animal Protein, high red meat, 2050	HEP High Animal Protein, Low red meat, 2011	HEP High Animal Protein, Low red meat, 2050	HHEP vegetarian (no eggs), 2011	HHEP vegetarian (no eggs), 2050
Animal Protein	2.90	4.06	5.85	8.19	3.47	4.85	-	-
Fish Protein	0.38	0.53	0.34	0.47	0.34	0.47	-	-
Plant Protein	0.12	0.17	0.22	0.30	0.30	0.42	1.58	2.21
Milk	0.64	0.89	0.59	0.83	0.59	0.83	0.59	0.84
Grains	0.88	1.24	0.54	0.76	0.64	0.90	0.54	0.75
Fruits & Vegetables	0.32	0.44	0.58	0.82	0.58	0.82	0.58	0.81
Oil and Fat	0.07	0.10	0.03	0.04	0.03	0.04	0.03	0.04
Sugar	0.04	0.05	-	-	-	-	-	-
Other	0.29	0.41	0.24	0.34	0.35	0.49	0.24	0.34
**Total GHGs**	**5.64**	**7.89**	**8.39**	**11.74**	**6.30**	**8.82**	**3.56**	**4.99**
Percent contribution of protein to total CF based on different scenarios								
Animal Protein (Includes eggs)	52%	52%	70%	70%	55%	55%	0%	0%
Fish Protein	7%	7%	4%	4%	5%	5%	0%	0%
Plant Protein	2%	2%	3%	3%	5%	5%	44%	44%

*Animal Protein includes eggs

#High animal protein is based on 65% of protein servings being from animal sources, which is based on the proportion of FAO 2011 production of animal protein

&Low animal protein is based on 25% of protein servings being from animal sources

High red meat is 7 servings per week based on 2011 production of red meat

Low red meat is 2 servings per week based on HHEP recommendations
